# Effects of Different doses of Silk Peptide on Energy Metabolism During Exercise in Mice

**DOI:** 10.20463/jenb.2017.0056

**Published:** 2017-03-31

**Authors:** Jisu Kim, Jonghoon Park, Bokyung Kim, Chi-Ho Lee, Kiwon Lim, Heajung Suh

**Affiliations:** 1 Physical Activity and Performance Institute (PAPI), Konkuk University, Seoul Republic of Korea; 2 Department of Physical Education, Korea University, Seoul Republic of Korea; 3 Department of Medicine, Konkuk University, Chungju Republic of Korea; 4 Department of Food Science and Biotechnology of Animal Resources, Konkuk University, Seoul Republic of Korea; 5 Department of Physical Education, Konkuk University, Seoul Republic of Korea

**Keywords:** Silk peptide, Energy metabolism, Exercise, Dose-response

## Abstract

**[Purpose]:**

This study was carried out to determine the optimal dose of silk peptide for enhancing fat metabolism during exercise.

**[Methods]:**

Fifty male ICR mice were randomly divided into five groups: Sed, SP0, SP200, SP400, and SP800. All SP mice underwent training by running on a treadmill 5 times a week for 2 weeks (20 m/min, 8° slope, 50 min/day for the first week and 25 m/min, 8° slope, 50 min/day at about 70-75% of maximum oxygen uptake for the second week).

**[Results]:**

After the 2 weeks, fat oxidation was measured during a 1-h exercise at the training conditions of the second week and was found to be 1.02 ± 0.15, 1.04 ± 0.17, 0.98 ± 0.10, 1.14 ± 0.19, and 1.15 ± 0.07 g/kg/h for Sed, SP0, SP200, SP400, and SP800 groups, respectively. The SP800 group had significantly higher fat oxidation levels than the SP0 group did at 36, 40, and 56 min and the Sed group did at 2, 4, 6, 8, 12, 14, 16, 20, 40, 46, 50, 52, 56, and 60 min. However, there was no significant difference among the groups in carbohydrate oxidation during the 1-h exercise. SP doses of 200 mg/kg and 400 mg/kg did not show any effect on fat and carbohydrate oxidation.

**[Conclusion]:**

In conclusion, 800 mg/kg of silk peptide is the optimal dose for enhancing fat metabolism during exercise. In addition, silk peptide treatment could reduce body weight by enhancing fat metabolism.

## INTRODUCTION

Silk peptide (SP) is a natural biomolecule that has been used in powder or extract form for a variety of purposes in Asian countries^1, 2^. SP comprises biopolymers produced by silkworm cocoons for protection from the environment during metamorphosis to the mature moth stage^3^. Nowadays, SP is used in various fields such as biotechnology and biomedicine since it does not cause any side effects^4, 5^. 

Recently, in vitro and in vivo studies have shown could stimulate lipolysis and improve health and exercise performance^1, 6, 7^. In 2012, Lee et al^8^. reported that treatment with 1 mg/mL SP + 0.2 nM insulin increases glucose uptake (124 ± 2.5%) via upregulation of glucose transporter type 4 (GLUT4) and decreases fat accumulation via upregulation of leptin in 3T3-L1 preadipocytes. In addition, treatment with SP inhibited the differentiation of preadipocytes and adipogenesis by modulating the peroxisome proliferator-activated receptor alpha (PPAR-α) signal transduction pathway and decreased body weight and size of adipocytes (86.1 ± 2.5%) in a high-fat diet-fed animal model. Furthermore, the addition of 5% SP to normal diet reduced body weight and abdominal fat in rats. In conclusion, SP ingestion might reduce adipose tissue by both stimulating lipolysis and inhibiting lipogenesis. Moreover, 5 weeks of SP treatment with swim training increased fat oxidation via upregulation of adenosine monophosphate-activated protein kinase (AMPK) and PPAR-α in liver cells^9^. The weights of abdominal and epididymal fat pads were lower in animals receiving SP treatment along with swim training than that in untreated animals undergoing swim training only, i.e., SP intake and/or swimming could activate fat metabolism. 

Recently, we used an open circuit calorimetry system to investigate the effects of SP administration on energy expenditure and substrate utilization in resting mice for 24 h. We found that the administration of SP during 2 weeks of endurance training (70% of maximum oxygen uptake) increased fat oxidation by about 16% compared to that reported for the group (not receiving SP)^10^. Interestingly, we found that the maximum oxygen uptake significantly increased after treatment with 800 mg/kg SP for 2 weeks. Moreover, fat oxidation during a 1-h exercise was 13% higher in the SP-treated (SP + endurance training) group than that in the non-SP-treated (endurance training only) group. These results suggest that SP could be an effective supplement for enhancing fat metabolism when used in combination with endurance training. However, 800 mg/kg SP is a large amount of worm protein to be consumed by humans (around 50 g needed for a 60-kg person). In addition, it has still not been elucidated whether SP treatment along with endurance training could enhance fat metabolism during exercise in a dose-dependent manner. 

Accordingly, the aim of the present study was to determine the optimal SP dose for enhancing fat metabolism during exercise. This was achieved by investigating the effects of different SP doses (200, 400, and 800 mg/kg) on energy metabolism during exercise using the open circuit calorimetry system. 

## METHODS

### Animals

**Figure 1.**
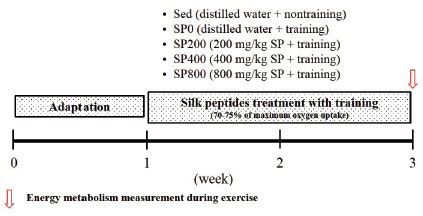
Experimental design.

Fifty male ICR mice (6 weeks old) were obtained from Orient Bio Inc. (Seongnam, Korea). All mice were housed in standard plastic cages (1643 × 766 × 1894 m/m; 5 mice/cage) under controlled conditions of humidity (50%) and temperature (23 ± 1 °C) with alternating 12-h light/dark cycles. They were adapted to the laboratory housing conditions for 7 days, and given free access to water and a non-purified commercial diet (5L79, Orient Bio Inc.) containing crude protein, 180 g/kg diet; crude fat, 52 g/kg diet; crude fiber, 52 g/kg diet; minerals, 57 g/kg diet; and carbohydrates, 368 g/kg diet. The protein, fat, and carbohydrate ratio (%) based on calories was 21:14:65, and the gross and metabolizable caloric contents of the diet were 4.04 and 3.21 kcal/g, respectively. Details of the experimental design are shown in Figure 1. 

Mice were randomly divided into 5 groups; Sed (distilled water), SP0 (distilled water, 0 mg/kg SP, no training), SP200 (200 mg/kg SP + training), SP400 (400 mg/kg SP + training), and SP800 (800 mg/kg SP + training). All SP groups (SP0, SP200, SP400, and SP800) underwent training by running on a treadmill 5 times a week for 2 weeks. SP was dissolved in distilled water and administered to the SP groups orally intraperitoneally 1 h before the endurance training. The Sed and SP0 groups received the vehicle (distilled water) only. 

### Silk peptide

The SP was obtained from Worldway Co., Ltd (Jeoneui, Korea). It is mainly composed of alanine (34.36%), glycine (27.23%), isoleucine (15.51%), serine (9.58%), and minor amounts of other amino acids. The detailed composition of SP is shown in Table 1. The molecular weight of SP ranges from 150 D to 350 D with an average molecular weight of about 250 D. SP was dissolved in distilled water and administered to the SP200, SP400, and SP800 groups; while the Sed and SP0 groups were administered distilled water orally every day for 2 weeks^2, 10^. 

**Table 1:** Amino acid compositions (%) of SP

Amino acid			
Ala	34.36	Phe	0.87
Gly	27.23	Pro	0.44
Iso	15.51	Tyr	0.41
Ser	9.58	His	0.21
Val	3.49	Arg	0.17
Thr	2.00	Met	0.10
Asp	1.68	Lys	0.10
Glu	1.28	Cys	0.05
Ile	1.25	Trp	0.05
Leu	1.24	Sum	100.00

### Training method

All mice were adapted to a treadmill training intensity of 15 m/min, 8° slope for 3 days. The mice were then tested 5 times per week for 2 weeks at the following training conditions: 20 m/min, 8° slope, 50 min/day for the first week and 25 m/min, 8° slope, 50 min/day (about 70-75% of maximum oxygen uptake) for the second week^2, 10, 11^. 

### Energy metabolism alterations during exercise

After 2 weeks of training, energy metabolism was measured during a 1-h exercise at the training conditions of the second week (25 m/min, 8° slope, 70-75% of maximum oxygen uptake). Mice were placed in exercise metabolism chambers for adaptation 2 h before the measurement^10, 12, 13^. 

### Statistical analysis

Data are given as mean ± standard deviation (SD). All statistical analyses were performed with SPSS version 19.0 software (SPSS, Inc., Chicago, IL, USA). Oxygen uptake, carbon dioxide production, RER (respiratory exchange ratio), carbohydrate oxidation, fat oxidation, food intake, and body weight were analyzed by twoway repeated measures analysis of variance (ANOVA). One-way ANOVA was used to determine the changes in energy metabolism during exercise and Bonferroni post-hoc analysis was conducted if significance was obtained. Differences were considered significant at P < 0.05. 

## RESULTS

### Changes in body weight and food intake

Table 2 shows the changes in body weight and food intake in Sed, SP0, SP200, SP400, and SP800 groups after 2 weeks of SP treatment and endurance training. There were no significant differences between the groups in the final body weights (38.82 ± 1.6, 37.7 ± 1.4, 38.1 ± 1.7, 37.6 ± 1.5, and 37.7 ± 2.0 g) and weight gain (2.9 ± 0.6, 2.30 ± 1.8, 2.52 ± 0.8, 2.54 ± 1.6, and 2.44 ± 1.3 g). Nevertheless, food intake (in g/day and g/2 weeks) was significantly higher in the SP800 group than in the Sed, SP0, SP200, and SP400 groups. 

**Table 2:** Body weight and food intake changes for 2 weeks treatment Sed, SP0, SP200, SP400 and SP800 groups

BW	Sed	SP0	SP200	SP400	SP800
Initial (g)	35.65 ± 1.2	35.42 ± 1.4	35.64 ± 1.4	35.13 ± 1.7	35.26 ± 1.6
Final (g)	38.82 ± 1.6	37.7 ± 1.4	38.1 ± 1.7	37.6 ± 1.5	37.7 ± 2.0
Gain (g)	2.9 ± 0.6	2.30 ± 1.8	2.52 ± 0.8	2.54 ± 1.6	2.44 ± 1.3
Food intake (g/day)	7.6 ± 1.1	7.9 ± 0.18	6.5 ± 0.4	7.4 ± 0.9	10.2 ± 3.1*
Food intake (g/2weeks)	98.2 ± 10.7	96.3 ± 0.0	87.6 ± 0.2	100.5 ± 1.3	133.7 ± 2.8*
FER	0.38 ± 0.0	0.23 ± 0.2	0.34 ± 0.1	0.38 ± 0.2	0.37 ± 0.1

The values are presented as means ± standard deviations.

* vs. all the other groups, p<0.001.

### Energy metabolism during exercise

**Figure 2.**
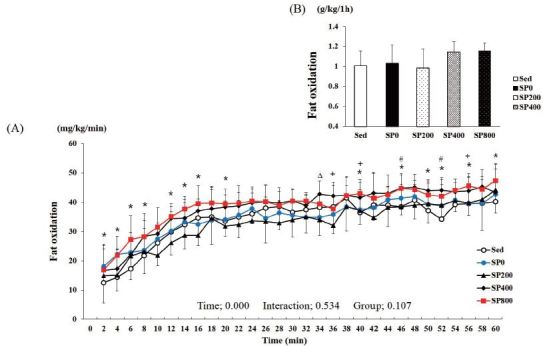
**Change in the fat oxidation level during a 1-h exercise (A). The sum of the fat oxidation level during a 1-h exercise (B).** Sed: distilled water, SP0: distilled water with training, SP200: 200 mg/kg SP with training, SP400: 400 mg/kg SP with training, SP800: 800 mg/kg SP with training. # Sed vs. SP400, P < 0.05; * Sed vs. SP800, P < 0.05; Δ SP0 vs. SP400, P < 0.05; + SP0 vs. SP800, P < 0.05. Values are presented as means ± standard deviations (n = 40).

Fat oxidation during the 1-h exercise was calculated from the carbon dioxide production (VCO2) and oxygen consumption (VO2) values. Two-way ANOVA with repeated measures for fat oxidation showed that time had a signiﬁcant effect (P < 0.001) on fat oxidation, while group (P = 0.107) and group-by-time interactions (P = 0.534) did not (Figure 2 A). The levels of fat oxidation during the 1-h period in the Sed, SP0, SP200, SP400, and SP800 groups were 1.02 ± 0.15, 1.04 ± 0.17, 0.98 ± 0.10, 1.14 ± 0.19, and 1.15 ± 0.07 g/kg/h, respectively. Fat oxidation in the SP800 group was 13 and 11% higher than that in the Sed and SP0 groups, respectively (Figure 2 B). When investigating fat oxidation at certain time points, it was found to be significantly higher in the SP800 group than that in the SP0 group at 36, 40, and 56 min and the Sed group at 2, 4, 6, 8, 12, 14, 16, 20, 40, 46, 50, 52, 56, and 60 min. However, fat oxidation was significantly higher in the SP400 group than that in the SP0 group at 34 min only and the Sed group at 46 and 52 min. 

**Figure 3.**
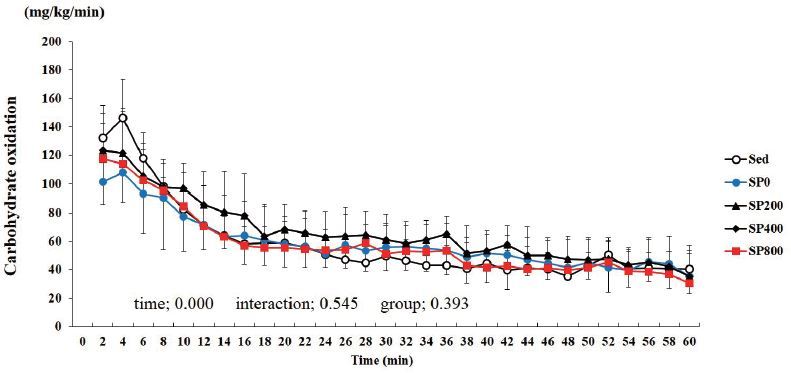
**Change in the carbohydrate oxidation level during a 1-h exercise.** Sed: distilled water, SP0: distilled water with training, SP200: 200 mg/kg SP with training, SP400: 400 mg/kg SP with training, SP800: 800 mg/kg SP with training. Values are presented as means ± standard deviations (n = 40).

Two-way ANOVA with repeated measures for carbohydrate oxidation showed signiﬁcant time effect (P < 0.001), but not for group (P = 0.393) and group-bytime interactions (P = 0.545) (Figure 3). Regarding carbohydrate oxidation, there was no significant difference among the groups during the 1-h exercise. 

## DISCUSSION

In the present study, we used an open circuit calorimetry system to investigate the effect of different doses of SP (200, 400, and 800 mg/kg) on energy metabolism during a 1-h exercise in mice. We found that treatment with 800 mg/kg SP for 2 weeks together with endurance training enhanced fat oxidation during a 1-h exercise in the early (until 20 min after the start) and late (around last 20 min) phases. However, the total amount of fat oxidation during the 1-h period did not reach a statistically significant level (fat oxidation in the SP800 group was 13 and 11% higher than that in the Sed and SP0 groups, respectively). However, lower doses of SP (200 and 400 mg/kg) had little effect on fat oxidation in mice undergoing training. 

We previously reported that fat oxidation during a 1-h exercise in the SP group (800 mg/kg SP + endurance training for 2 weeks) was 13% higher than that in the untreated training group^2^. This result was similar to the result of the present study, which demonstrated an 11% increase in fat oxidation in the SP-treated group compared to that reported for the untreated group. However, we found that lower doses of SP (200 and 400 mg/kg) had no effect on fat metabolism during exercise. Thus, we concluded that 800 mg/kg of SP could be effective for training athletes such as long-distance runners. 

Interestingly, we observed that daily food intake (g/day) was markedly higher in the SP800 group than that in the other groups although the final body weight and body weight gain did not differ among groups. A recent study reported that long-term (8 weeks) administration of SP along with high-fat diet (lard content; 20.69%) reduced body weight and body fat although food intake did not differ between the groups^1^. In another study, administration of SP for 5 weeks with swimming exercise decreased body weight and body fat to a greater extent than that observed with swimming only^9^. According to the results from Lee et al (2012)^1^, the decreased fat accumulation is mediated by upregulation of leptin in 3T3-L1 preadipocytes. The results of our study demonstrated that the SP800 group appeared to burn much more fat while doing physical activity (running). Thus, we cautiously assumed that mice treated with 800 mg/kg SP might utilized more energy during the dark cycle (physical activity period) as well as during training. 

However, the mechanism by which SP intake (800 mg/kg) further enhanced fat oxidation and showed slight anti-obesity effect with exercise is still unclear. In addition, the dose of 800 mg/kg body weight of SP would be a very large amount of worm protein intake per day for human subjects. Thus, further studies are required to elucidate the molecular mechanisms related to the anti-obesity effect of SP and to search for strategies to reduce the amount of SP intake, e.g., the combination of SP with other non-protein supplements to increase fat metabolism. 

In conclusion, our results suggest that 800 mg/kg of SP could be the optimal dose for enhancing fat metabolism in combination with endurance training in mice. In addition, SP treatment was found to be effective in reducing body weight by enhancing fat metabolism. However, further studies are required to elucidate the mechanisms underlying the SP anti-obesity effect and to determine the suitable dose of SP for enhancing fat metabolism in human subjects. 

## COMPETING INTERRESTS

The authors declare that they have no competing interests. 

## References

[1] Lee SH., Park GY., Bae DK., Yang YH. (2012). Silk and silkworm pupa peptides suppress adipogenesis in
preadipocytes and fat accumulation in rats fed a high-fat
diet. *Eur J Nutr*.

[2] Kim JS., Hwang HJ., Park JH., Yun HY., Suh HJ., Lim KW. (2014). Silk peptide treatment can improve the exercise performance of
mice. *J Int Soc Sports Nutr*.

[3] Seo CW., Um IC., Rico CW., Kang MY. (2011). Antihyperlipidemic and body fat-lowering effects of silk proteins
with different fibroin/sericin compositions in mice fed with high fat
diet. *J Agric Food Chem*.

[4] Mora CM., Mrowiec A., Maria GV., Atonia A., Jose LC., Francisco JN. (2012). Fibroin and sericin from Bombyx mori silk stimulate cell
Migration through upregulation and phosphorylation of c-Jun. *PLoS ONE*.

[5] Kim JS., Park JH., Lim KW. (2016). Nutrition Supplements to Stimulate Lipolysis: A Review in
Relation to Endurance Exercise Capacity. *J Nutr Sci Vitaminol*.

[6] Shin S., Yeon S., Park D., Oh J., Kang H., Kim S., Joo SS., Lim WT., Lee JY., Choi KC., Kim KY., Kim SU., Kim JC., Kim YB. (2010). Silk amino acids improve physical stamina and male reproductive
function of mice. *Biol Pharm Bull*.

[7] Shin SH., Park DS., Yeon SH., Jeon JH., Kim TK., Joo SS., Lim WT., Lee JY., Kim YB. (2009). Stamina-enhancing effects of silk amino acid preparations in
mice. *Anim Res*.

[8] Lee SH., Park GY., Bae DK., Yang YH. (2012). Silk and silkworm pupa peptides suppress adipogenesis in
preadipocytes and fat accumulation in rats fed a high-fat
diet. *Eur J Nutr*.

[9] Ryu SP. (2014). Silkworm pupae powder ingestion increases fat metabolism in
swim-trained rats. *J Exerc Nutrition Biochem*.

[10] Kim JS., Hwang HJ., Yun HY., Kim BK., Lee CH., Suh HJ., Lim KW. (2013). Silk peptide intake increases fat oxidation at rest in exercised
mice. *J Nutr Sci Vitaminol*.

[11] Hwang HJ., Kim JS., Park JH., Yun H., Cheon WK., Kim BK., Lee CH., Suh HJ., Lim KW. (2014). Red ginseng treatment for two weeks promotes fat metabolism
during exercise in mice. *Nutrients*.

[12] Jeon YR., Kim JS., Hwang HJ., Lim KW. (2012). Effects of endurance training for 4weeks on resting metabolic
rate and excess post-exercise oxygen consumption in mouse. *J Exerc Nutr Biochem*.

[13] Lim KW., Kim JS., Jeon YR., Hwang HJ., Suh HJ. (2011). Measurement of resting metabolic rate using metabolic chamber in
resting rats. *J Exerc Nutr Biochem*.

